# IAMSAM: image-based analysis of molecular signatures using the Segment Anything Model

**DOI:** 10.1186/s13059-024-03380-x

**Published:** 2024-11-11

**Authors:** Dongjoo Lee, Jeongbin Park, Seungho Cook, Seongjin Yoo, Daeseung Lee, Hongyoon Choi

**Affiliations:** 1 Portrai, Inc, 78-18, Dongsulla-Gil, Jongno-Gu, Seoul, 03136 Republic of Korea; 2 Department of Nuclear Medicine, Seoul National University Hospital, 03080 Seoul, Republic of Korea; 3 Department of Nuclear Medicine, Seoul National University College of Medicine, 03080 Seoul, Republic of Korea

**Keywords:** Spatial transcriptomics, Image segmentation, H&E image, Deep learning, Histology

## Abstract

Spatial transcriptomics is a cutting-edge technique that combines gene expression with spatial information, allowing researchers to study molecular patterns within tissue architecture. Here, we present IAMSAM, a user-friendly web-based tool for analyzing spatial transcriptomics data focusing on morphological features. IAMSAM accurately segments tissue images using the Segment Anything Model, allowing for the semi-automatic selection of regions of interest based on morphological signatures. Furthermore, IAMSAM provides downstream analysis, such as identifying differentially expressed genes, enrichment analysis, and cell type prediction within the selected regions. With its simple interface, IAMSAM empowers researchers to explore and interpret heterogeneous tissues in a streamlined manner.

**Supplementary Information:**

The online version contains supplementary material available at 10.1186/s13059-024-03380-x.

## Background

Spatial transcriptomics (ST) enables the analysis of gene expression patterns inside tissues while maintaining their spatial context [1]. However, researchers often encounter difficulties when working with ST data due to its complexity, high-dimensionality, spatial constraints, large data volumes, and the lack of user-friendly tools [1, 2]. For instance, clustering spots or cells within one or multiple ST libraries must exhibit spatial continuity for each cluster, which requires the use of a complex algorithm [3]. Furthermore, the manual process of identifying genes associated with specific regions, based on domain knowledge such as pathologist-labeled annotation, introduces a subjective analytic workflow [4, 5]. Integrating the interpretation of tissue image patterns, along with multidimensional molecular information, allows researchers to gain a deeper understanding of the pathophysiology within spatial contexts [6–8]. Therefore, an interactive and user-friendly interface for ST to analyze tissue images should also be developed to facilitate improved communication for basic researchers, clinicians, and bioinformaticians.

Here, we introduce IAMSAM (Image-based Analysis of Molecular signatures using the Segment Anything Model), a user-friendly web-based tool designed to comprehensively analyze ST data, enabling a better understanding of complex tissues by integrating images with molecular information. IAMSAM leverages the power of the “Segment-anything,” a state-of-the-art deep learning model developed by Meta [9], to identify regions of interest (ROIs) from tissue images in ST datasets. The SAM model exhibits exceptional performance, achieving real-time performance and efficiently utilizing computational resources. Moreover, it stands as the first foundation model for general image segmentation, providing interactive prompting capabilities. It has been specifically designed to address the problem of zero-shot image segmentation pre-trained on an extensive and diverse dataset consisting of over 1 billion masks derived from 11 million images, ensuring its robust performance. We used this model to handle various tissue images (e.g., H&E, DAPI, and immunofluorescence images), taking advantage of its effectiveness and adaptability to handle different image distributions and workloads through zero-shot or few-shot learning. This excellence leads to conducting various downstream analyses such as identifying differentially expressed genes (DEGs), enrichment analysis, and cell type prediction of user-selected regions. Moreover, the regions can be determined by image patterns rather than gene expression, providing an opportunity to analyze gene expression patterns and features based on image-based key patterns. In this study, we demonstrated the usage of IAMSAM with publicly available ST datasets. With its simple and accessible interface, IAMSAM enables researchers to explore and interpret their ST data user-friendly, which can lead to new insights into gene expression patterns associated with pathophysiology and potential biomarkers for diseases.

## Results

### Overview of IAMSAM

**Fig. 1:**
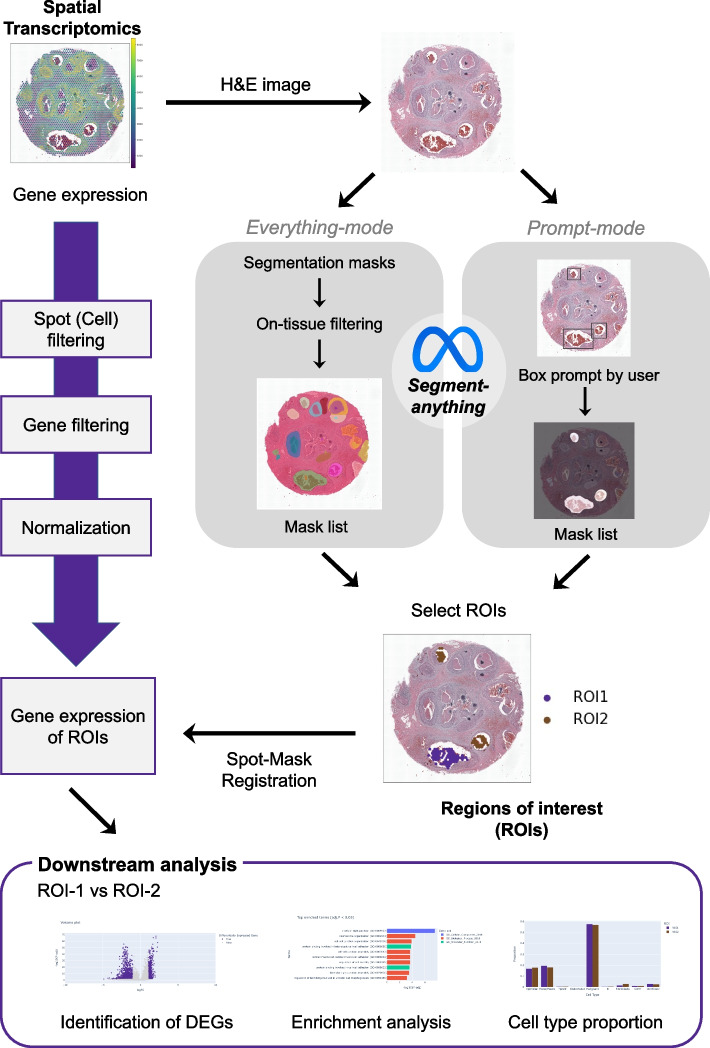
Workflow of IAMSAM. This figure provides an overview of the workflow of IAMSAM. The gene expression of ST data is preprocessed through spot filtering, gene filtering, and normalization step. The H&E image of the ST data is segmented using the SAM in two different modes: everything-mode and prompt-mode. The selected ROIs are then subjected to downstream analysis, which includes DEG identification, enrichment analysis, and cell type proportion analysis

IAMSAM is a web-based tool designed for analyzing ST data, based on a general-purpose image segmentation algorithm named “Segment-anything” (Fig. 1). It utilizes the SAM for H&E image segmentation, which allows for morphological guidance in selecting ROIs for users. IAMSAM offers users with two modes for running the SAM algorithm: everything-mode and prompt-mode. In the everything-mode, IAMSAM automatically generates segment masks based on morphological features along whole tissues. On the other hand, the prompt-mode allows users to draw rectangle boxes, which serve as input prompts for the SAM model. Afterwards, users have the option to select one or multiple masks for ROI 1 and ROI 2 from the mask lists before proceeding with downstream analysis. IAMSAM automatically extracts the gene expression profile from the chosen ROIs, identifying not only DEGs between the ROIs but also enriched functional terms associated with these DEGs. Furthermore, IAMSAM provides cell type estimation of the selected regions, which can help users gain valuable insights into the cellular composition and heterogeneity of the tissue.

### H&E image segmentation

**Fig. 2:**
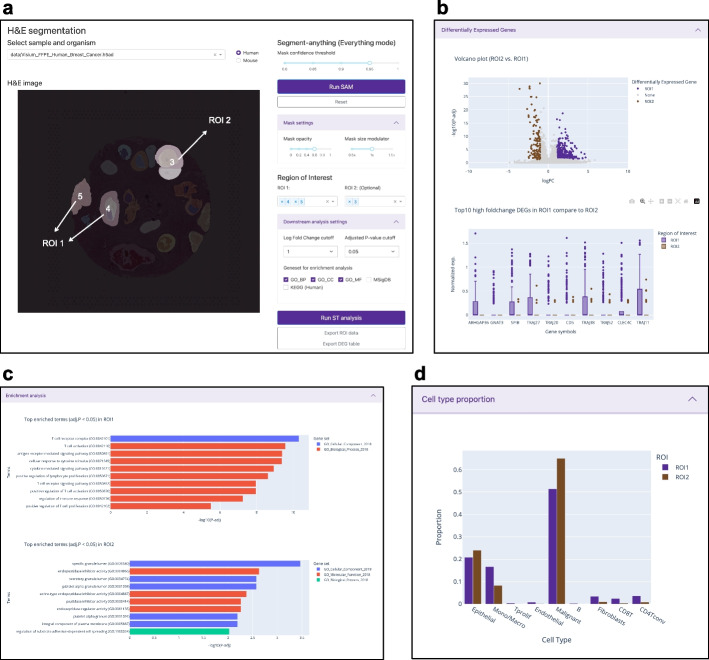
Overview of IAMSAM interface panels. **a** The main visualization panel displays the H&E slides of the ST data, along with the corresponding segmentation masks. These masks highlight different ROIs within the tissue image, allowing users to visually explore and select specific ROIs. After pressing “Run ST analysis,” the downstream analysis panel presents the results of downstream analysis, including (**b**) DEG analysis, **c** enrichment analysis, and **d** cell type proportion

Hematoxylin and eosin (H&E) are widely employed to observe tissue structure, distinguish different histological features, and are considered a gold standard in the field of histopathology [10]. Most ST platforms, particularly the 10x Visium platform, involve the inclusion of H&E staining and tissue imaging steps in the tissue preparation protocol [11]. This unique feature of the Visium platform allows IAMSAM to utilize the H&E image. When users select the samples to analyze on the dropdown menu, the H&E image of the sample appears in the main visualization panel (Fig. 2a). After configuring multiple parameters, such as mask confidence threshold, mask opacity, and mask size, users can click the “Run SAM” button to make inferences from the SAM. SAM takes the H&E slide images as input and creates a binary mask for each morphologically segmented region. IAMSAM visualizes these segment masks on the main visualization panel with a distinct palette, offering a user-friendly approach for researchers to analyze their ST data. Users can generate SAM masks and specify ROIs in two different modes, depending on their requirements or preferences. This approach not only reduces the time and effort required for manual annotation but also provides a more objective way of identifying morphological features and molecular signatures within the tissue.

### Downstream analysis

The following downstream analysis consists of three panels: identifying DEGs (Fig. 2b), enrichment analysis (Fig. 2c), and cell type proportion (Fig. 2d). As all the downstream plots are interactively made, the various convenient features including auto-scaling, manual scaling, zoom-in, zoom-out, capture, and the management of the coordinates are supported for each plot.

The first panel is the DEG module, which includes both the volcano plot and the box plot. The volcano plot represents the log-fold change on the *x*-axis, where positive values indicate up-regulation in the ROIs, and the statistical significance on the *y*-axis. Users can set the “logFC cutoff” and “p-adj cutoff” in the parameter panel (Fig. 2b). Genes that meet the criteria of having a fold change value exceeding the FC cutoff and an adjusted *p*-value less than the adjusted *p*-value cutoff are displayed in purple for ROI 1 and brown for ROI 2, while the remaining genes are shown in gray. The box plot, on the other hand, focuses on the top 10 genes selected from the up-regulated DEGs within the ROI 1. These genes are ranked based on their fold changes, reflecting the relative difference in expression levels between the ROI 1 and ROI 2.

In the second panel, IAMSAM performs over-representation analysis (ORA) on the DEGs identified in the selected ROIs. The goal of ORA is to assess whether specific gene sets or functional categories are overrepresented among the DEGs, indicating their potential involvement in specific biological processes or molecular functions. IAMSAM offers users a choice of gene sets for enrichment analysis, including three GO (Gene Ontology) terms (biological process, cellular component, and molecular function), as well as gene sets from MSigDB (Molecular Signatures Database) and KEGG (Kyoto Encyclopedia of Genes and Genomes). Users can select the gene sets of interest based on their preferences to perform the enrichment analysis. IAMSAM calculates the statistical significance of the enrichment terms and filters them based on adjusted p-values. Only the terms that demonstrate statistical significance, with adjusted *p*-values below 0.05, are displayed in the form of a bar plot. This visualization allows users to easily identify the enriched terms and gain insights into the functional annotations associated with the DEGs.

For the last panel, IAMSAM provides cell type proportion within the selected ROIs. We exploit CellDART [12] to annotate Visium data with reference scRNAseq data by default, but users can also choose other cell-type deconvolution algorithms in the preprocessing step. The proportions of cell types are visualized as a bar chart, displaying the differences between ROI 1 and ROI 2 for clarity and simplicity. This concise representation offers a clear overview of the predominant cell types present in the tissue sample and aids in understanding the cellular composition within the spatial context.

### Two modes of IAMSAM: everything-mode and prompt-mode

In the everything-mode, users can obtain segmented masks for the entire tissue image by simply clicking the “Run SAM” button. IAMSAM automatically segments the entire image, creating masks that distinguish various morphological features or regions within the tissue without requiring any additional prompts.

**Fig. 3:**
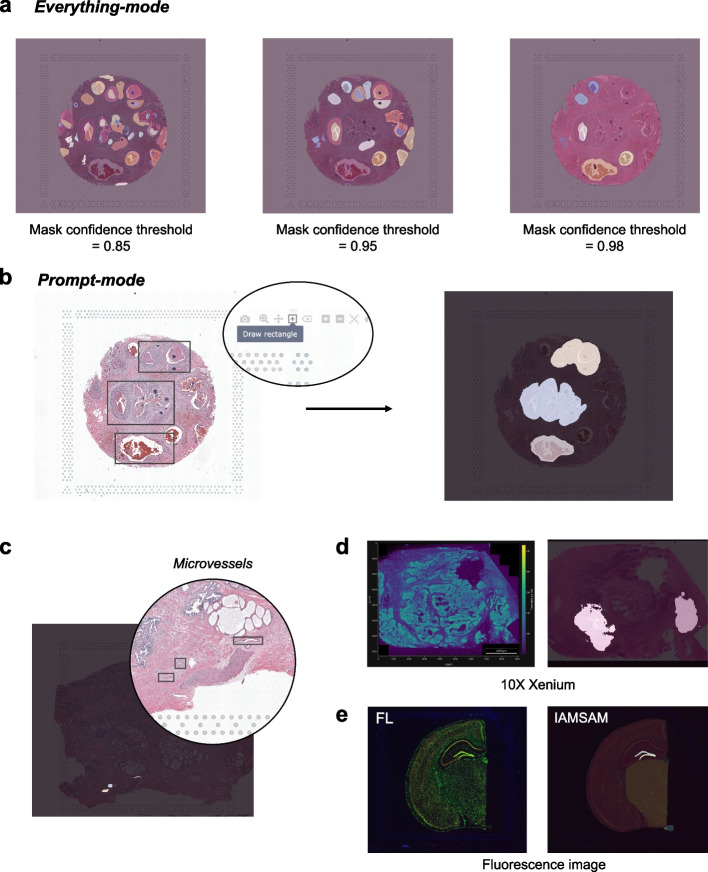
Main characteristics of IAMSAM. This figure introduces the two main modes of operation in IAMSAM: everything-mode and prompt-mode. **a** In the everything-mode, IAMSAM generates segmentation masks for the entire tissue images. The mask confidence threshold directly affects the segmentation result, where a higher threshold leads to more precise segmentation but fewer selected masks. **b** In the prompt-mode, users can provide prompts to the SAM model by drawing rectangle boxes on the visualization panel using the drawing tool provided by Plotly. When users input three rectangle boxes as drawn, IAMSAM returns the corresponding ROIs. **c** By combining the zoom-in interface with the prompt-mode, IAMSAM allows for the detailed examination of microscopic histology features, enhancing analysis capabilities. **d** IAMSAM can also process data from platforms like Xenium, following appropriate preprocessing steps. **e** IAMSAM is applicable to various imaging modalities, including fluorescence imaging, thereby expanding its utility in different experimental settings

The “Mask confidence threshold” parameter (Fig. 3a) is a crucial factor for users to consider because it determines the threshold value used to decide whether a predicted object or region in an image is considered a true positive or not. It is described as an intersection-over-union (IOU) score in the original literature, which is a metric used to measure the overlap between the predicted segmentation mask and the ground truth mask during training [9]. By increasing the threshold value, the model becomes more stringent in accepting predicted masks. This means that only masks with a higher predicted IOU value, indicating better quality and accuracy, will be included in the final segmentation results. Consequently, the number of selected masks may decrease. Conversely, reducing the threshold makes the model more permissive in accepting masks, even if their predicted IOU is low. This relaxation of criteria can yield a higher number of masks, including those with potentially lower quality. Users should control the balance between the number of masks and their quality in the segmentation results, based on their specific requirements and preferences.

After the segmentation, masks that do not contain any spots are filtered out, and the remaining masks are numbered in descending order based on their respective areas. Users can choose the mask number from a dropdown menu to assign masks as ROI 1 or ROI 2. Alternatively, they can directly click on the masks in the main visualization panel. This feature is enabled through the interactive interface of Plotly [13], which allows users to visualize the segmented regions and select the ROIs with ease. For an improved user experience, we have also added a feature that allows users to deselect a selected mask by simply re-clicking on it. If users want to perform a one-versus-others analysis, they can leave ROI 2 empty.

After all ROIs have been chosen, users can run downstream analysis on the ROIs with the “Run ST Analysis” button. By enabling users to select the masks of interest through a simple click, IAMSAM streamlines the analysis of ST data and allows researchers to quickly identify relevant cell types and gene expression patterns in their samples.

IAMSAM offers another mode called prompt-mode, which provides users with the flexibility to manually define the desired segments using rectangle boxes. This mode utilizes the prompt-input method of the original SAM algorithm, allowing users to specify boxes in the image that correspond to the objects they want to segment. Before running SAM, users can easily draw rectangles on the main visualization panel using the default rectangle drawing tool (Fig. 3b). Users can also conveniently track the number of boxes added and have the button to reset if any mistakes are made. Since box prompts are available in advance before running SAM, IAMSAM can run SAM in a batched manner, generating corresponding masks for multiple boxes simultaneously. If needed, users can utilize the zoom feature provided by Plotly when selecting ROIs in the prompt-mode. Upon clicking “Run SAM”, one or more masks are interpreted as the user's areas of interest, and subsequent downstream analysis is performed in the same way as the everything-mode (Additional file 1: Fig. S1).

### Versatility and expanded capabilities of IAMSAM

To uncover microscopic histological features, the prompt-mode in IAMSAM can be particularly powerful, especially when used with magnification. When applying IAMSAM to human prostate cancer Visium data, we demonstrated its capability to identify and select microvessels as ROIs using the prompt-mode and the zoom-in interface (Fig. 3c, Additional file 1: Fig. S2). Zooming in on specific tissue areas helped identify microvessels, which may not be readily apparent on a larger scale. Furthermore, our analysis revealed that pan-endothelial cell markers, such as *CAV1* (log FC = 3.21, − log10 P-adj = 2.93), *CAV2* (log FC = 2.14, − log10 P-adj = 1.56), and *CAVIN1* (log FC = 1.50, − log10 P-adj = 1.94), were up-regulated within the ROIs. In line with these findings, a GO term related to “focal adhesion,” specific to endothelial cells, was enriched, indicating the involvement of endothelial cells in these ROIs [14]. We also validated these microvessel areas with pathologists to ensure the accuracy of our identification.

Although IAMSAM is designed for analyzing Visium data, it can also process image-based ST technologies like Xenium and MERSCOPE if proper preprocessing steps are executed. Expanding IAMSAM to include image-based ST data allows for a broader range of applications and greater flexibility in analyzing different types of ST datasets. We demonstrated the capability of IAMSAM to analyze Xenium data using the publicly available Xenium human colon cancer dataset. If a post-Xenium H&E image is available, users can preprocess Xenium data with affine transformation and resizing (Fig. 3d, Additional file 1: Fig. S3). This expansion enhances the versatility of IAMSAM, making it a powerful tool for integrating and analyzing ST data from various sources. Lastly, we explored the application of IAMSAM with an optical image different from H&E staining (Fig. 3e). We utilized a combined image of three distinct color channels corresponding to DAPI (4′,6-diamidino-2-phenylindole), anti-GFAP, and anti-NeuN staining. In this case, the successful identification of the dentate gyrus (DG) structure demonstrated the versatility and feasibility of IAMSAM in handling different imaging modalities. This finding further solidifies SAM as a general image segmentation algorithm that can be applied across various experimental setups. This feature highlights the broad applicability of IAMSAM and its potential to provide valuable information from diverse imaging modalities that spatially correspond to ST data [15].

### Characterizing spatial tumor heterogeneity in a breast *cancer* sample using IAMSAM

**Fig. 4:**
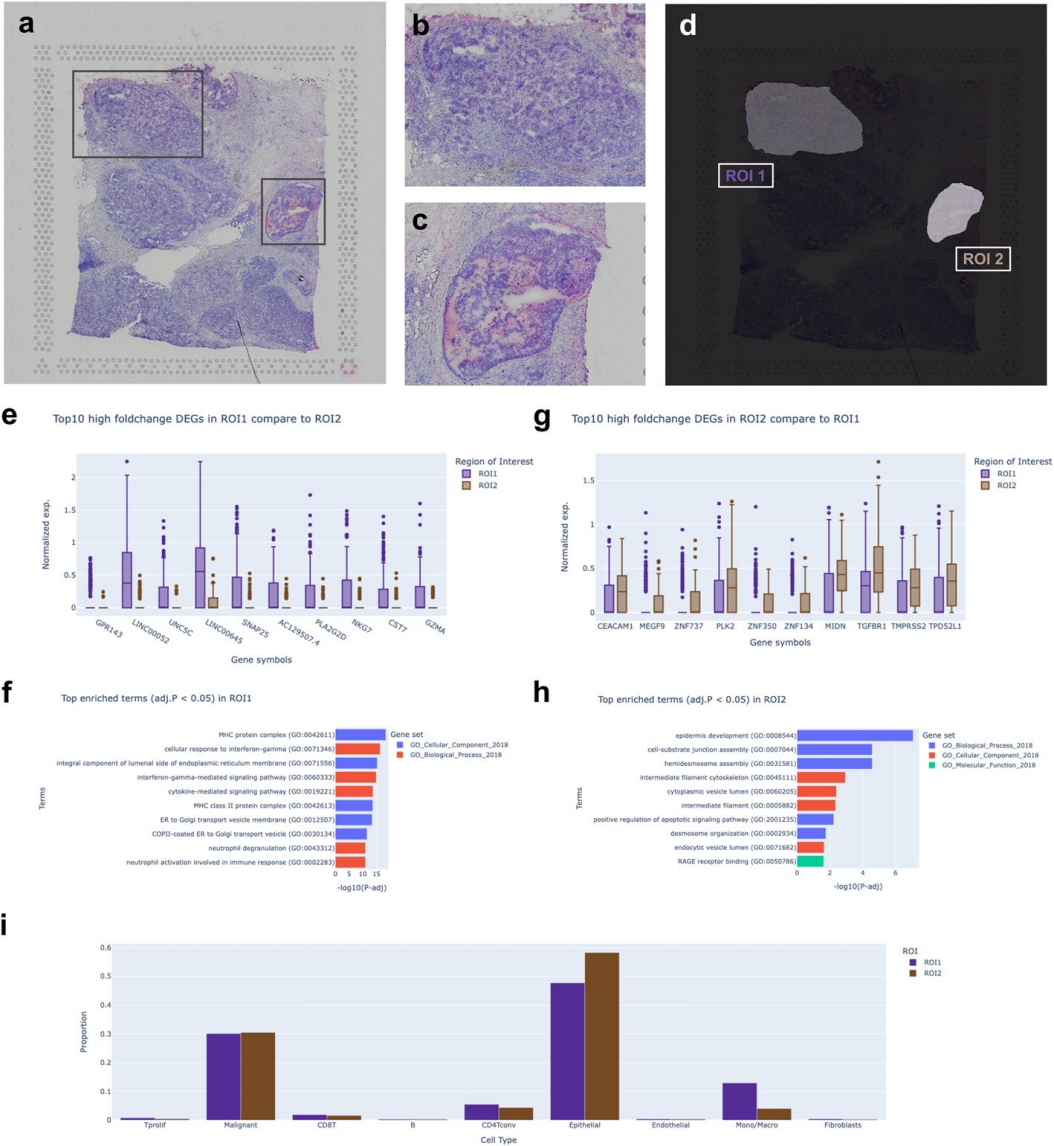
Analysis of cancer heterogeneity in human breast cancer using IAMSAM. **a** H&E-stained image of the human breast cancer block A Sect. 1.1 dataset, showing the selected ROIs. **b** Close-up image of ROI 1, highlighting distinct morphological features. **c** Close-up image of ROI 2, highlighting distinct morphological features. **d** IAMSAM analysis showing the identified ROIs based on distinct morphological features. **e** Box plot showing the top 10 high fold change DEGs in ROI 1 compared to ROI 2. **f** Bar plot of the top enriched GO terms (adjusted *p*-value < 0.05) in ROI 1. **g** Box plot showing the top 10 high fold change DEGs in ROI 2 compared to ROI 1. **h** Bar plot of the top enriched GO terms (adjusted *p*-value < 0.05) in ROI 2. **i** Cell type proportion analysis showing the distribution of cell types within ROI 1 and ROI 2

To demonstrate an example of IAMSAM to discover the finding by integrating ST with morphological features, we inspected cancer heterogeneity within a Human breast cancer block A Sect. 1.1 dataset. We selected two ROIs (Fig. 4a–d) based on distinct morphological features observed in the dataset as an automatic method for delineating morphologically characteristic regions based on IAMSAM. Notably, ROI1 is identified as an invasive region, while ROI2 is classified as a ductal carcinoma in situ (DCIS) portion according to the pathological annotation of the previous literature [16]. The IAMSAM analysis identified ROI 1 as primarily characterized by immune-related processes compared to ROI 2. Differential gene expression analysis identified top genes such as *PLA2G2A*, *GPR143*, *LINC00052*, *UNC5C*, and *PLA2G2D* as significantly upregulated in ROI 1 compared to ROI 2 (Fig. 4e). Enrichment analysis revealed terms such as MHC protein complex, cellular response to interferon-gamma, and cytokine-mediated signaling pathway (Fig. 4f). These enrichments suggest a significant presence of immune cell infiltration and activity within ROI 1. The cell type proportion analysis further supported this, showing a high presence of immune cells such as monocytes/macrophages and CD4 T-cells (Fig. 4i). These findings align with the characteristics of invasive ductal carcinoma (IDC), where immune interactions are progressed compared with DCIS [17, 18]. In contrast to ROI 1, genes such as *CEACAM1*, *TGFBR1*, *ZNF737*, and *PLK2* were significantly upregulated in ROI 2 (Fig. 4g). The enriched GO terms for ROI 2 included epidermis development, cell-substrate junction assembly, and hemidesmosome assembly, which are indicative of epithelial processes and cell adhesion (Fig. 4h). The cell type proportion analysis revealed a predominance of epithelial cells and malignant cells, consistent with the features of the tumor core where malignant cells are predominant and exhibit strong epithelial characteristics (Fig. 4i). This result aligns with previous histological annotations [16], which identified ROI2 as the tumor region of DCIS and ROI1 as the invasive region of breast cancer. Beyond identifying distinct molecular and cellular characteristics within ROIs, IAMSAM can extend its utility by integrating with advanced bioinformatics tools for further analyses. For example, users can employ tools such as stLearn [5] to analyze cell–cell communication within ROIs selected by IAMSAM. This integration allows for the identification of top-scored ligand-receptor pairs for each ROI, providing insights into the molecular interactions within specific tissue regions (Additional file 1: Fig. S4 b). Additionally, ROIs can be inspected using compositional frameworks like TACCO [19], calculating distances from the ROI and illustrating changes in cell type deconvolution along these distances (Additional file 1: Fig. S4 c–d). This compatibility facilitates seamless integration with other tools, enabling more comprehensive and advanced analyses for researchers.

### Workflow advantages of IAMSAM over traditional methods in analyzing spatial heterogeneity

**Fig. 5:**
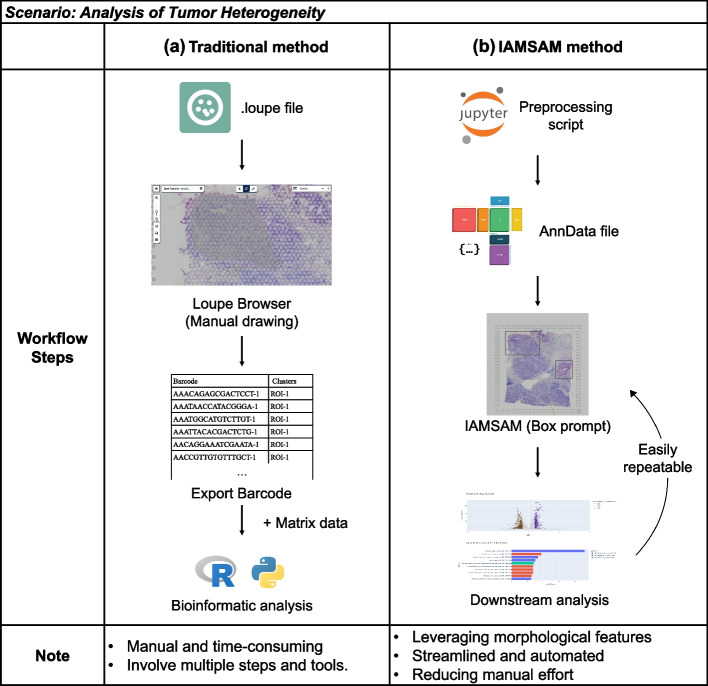
Comparative performance analysis of traditional method and IAMSAM method. **a** Traditional method involves manual drawing of ROIs in Loupe Browser, exporting barcode data, and performing downstream bioinformatic analysis using R or Python. This process is manual, time-consuming, and involves multiple steps and tools. **b** IAMSAM method utilizes a preprocessing script to create an AnnData file, followed by automated ROI identification and downstream analysis within the IAMSAM framework. This method leverages morphological features, is streamlined and automated, reducing manual effort and increasing reproducibility

The workflow of IAMSAM in analyzing the spatial morphological heterogeneity surpasses that of traditional methods, as illustrated in Fig. 5. Traditional methods involve a manual and time-consuming workflow, requiring multiple steps and tools. Typically, for Visium data, a loupe file is examined using Loupe Browser, where ROIs are manually drawn from scratch (Fig. 5a). This manual process is time-consuming and highly dependent on the analyst’s skill and consistency, leading to variability and reproducibility issues due to human error and subjective judgment. IAMSAM addresses this gap by automating the identification of ROIs using advanced image processing techniques that leverage morphological features (Fig. 5b). The use of box prompting in IAMSAM simplifies the process and ensures consistency, allowing multiple inspections of various regions. This automation eliminates the need for manual intervention, significantly reducing the time required for ROI identification and improving reproducibility. Additionally, traditional workflows often involve multiple disjointed steps and tools, such as exporting barcode tables, matching with matrix data, and performing separate downstream analyses using R or Python. This fragmentation is inefficient and prone to errors, as each step requires separated code, increasing the overall processing time and introducing potential points of failure. IAMSAM addresses this gap by providing a seamless, end-to-end workflow that integrates data preprocessing, ROI identification, and downstream analysis within a single platform. This streamlined workflow highlights the efficiency and accuracy of IAMSAM, making it a superior alternative to traditional methods for inspecting morphological heterogeneity and spatial patterns of the tissue, especially in tumor heterogeneity. The reduction in analysis time and manual intervention not only enhances productivity but also improves the consistency and reliability of the results, making IAMSAM an invaluable tool for cancer research.

## Discussion

Integrating SAM into ST libraries has shown great promise as a new workflow to enhance the interpretation of image-based characteristics in ST data analysis, leading to a more comprehensive molecular interpretation. This integration allows for accurate segmentation of histologically distinct structures and produces segments that align closely with gene expression clusters. By bridging the gap between histological features and molecular information, IAMSAM enables a comprehensive analysis of ST data. One of the strengths of IAMSAM lies in its user-friendly nature. This application has the potential to facilitate ST research by providing real-time and interactive tools for acquiring gene signatures from ST libraries. By lowering the barriers to entry in the field of ST, IAMSAM enables researchers to explore and analyze spatially resolved transcriptomic data more effectively.

While IAMSAM offers powerful analysis capabilities, users must consider several factors to utilize the tool effectively. First, it is crucial to select the proper mode when using IAMSAM. Our observations indicate that specific tissues may be better suited for the prompt-mode in IAMSAM, as this mode allows for capturing subtle and local differences that the everything-mode may overlook. As demonstrated by several use cases, we have significantly improved the segmentation performance by introducing an interactive function to IAMSAM. Second, users must select the proper preprocessing methods for analyzing their own data. Although IAMSAM provides example tutorials for preprocessing starting from the raw data, researchers should carefully consider variables like tissue type, species, and other pertinent characteristics. This decision guarantees reliable and precise downstream analysis. Despite the numerous advantages of IAMSAM in identifying meaningful regions of interest within ST data, some limitations warrant further discussion and future improvement. Firstly, IAMSAM currently does not support processing of multiple images. One of the primary motivations for computer-assisted ROI selection is to ensure reproducibility across different fields of view and datasets. However, the segmentation process with SAM could be varied in multiple images due to batch effects of images. This limitation underscores the need for developing and integrating unified processing capabilities of segmentation to enhance efficiency, especially in large-scale studies. In this regard, the stability of SAM's segmentation results may be influenced by variations in H&E staining intensity. Differences in staining intensity can impact the accuracy of segmentation, potentially leading to inconsistent results. Future improvements for IAMSAM should focus on enhancing the tool’s robustness to accommodate such variations. This could involve the implementation of normalization techniques or adaptive algorithms that adjust to staining intensity changes, ensuring more reliable segmentation outcomes. Exploring the impact of varying image dimensions and the number of images on segmentation performance can be a future direction for the applicability of this tool. Future developments should aim to assess and optimize the performance across a range of image sizes and dataset volumes.

Although IAMSAM has its limitations, it is true that the spatial context of single-cell omics studies can provide further insights into biology. Integrating this spatial information with diverse tissue images holds particular value, as it enhances the interpretability of the data [6]. In this regard, exploring the implications of visually discernible histological characteristics, such as dense cancerous areas or stroma-rich regions on images, becomes crucial. The critical role of IAMSAM is to elucidate the molecular characteristics associated with these distinctive image regions and determine which cells exhibit such image-specific characteristics through the integration of ST data and image data analysis. In essence, this approach allows for a comprehensive understanding of the molecular attributes underlying visually distinctive patterns in the images and the specific cellular contributions to these patterns. Moreover, ST analysis effectively reveals heterogeneity based on tissue image characteristics, extending beyond a mere assessment of cellular composition. IAMSAM presents a workflow that explains visually identifiable image features with molecular information, offering a new direction for ST analysis.

## Conclusions

IAMSAM is a user-friendly web-based tool designed to analyze ST data. The tool utilizes the SAM algorithm to segment H&E images of Visium data and performs statistical analysis to identify DEGs and their corresponding GO terms for each segmented region. With its simple and accessible interface, IAMSAM makes it easy for researchers to analyze and interpret their ST data. IAMSAM will be a valuable resource for researchers in the field of ST.

## Methods

### Dataset and preprocessing

We used four publicly accessible ST datasets, as shown in Fig. 4, to illustrate the utility of IAMSAM. These datasets were chosen to represent a variety of tissues and experimental setups, allowing for a thorough assessment of IAMSAM’s capabilities. We employed the Scanpy package [20] to perform initial data manipulation steps for preprocessing. Specifically, spots containing fewer than 200 transcripts were excluded from the feature matrix. Subsequently, a default log-normalization process was applied to each spot, facilitating the normalization and scaling of gene expression values across the dataset. For calculating cell type proportions, we used the CellDART [12] methods with default parameters. Additionally, the pixel coordinates in the ST images were adjusted by multiplying them with the scale factor to align the image coordinates with the corresponding spot positions in the dataset. The image was then cropped to focus on the tissue area, excluding fiducial spots, while minimizing the padding around the image. This cropped image was used as input for SAM.

### Segment Anything Model

IAMSAM uses the SAM to segment tissue images derived from ST data. SAM enables users to define ROIs effortlessly by detecting morphologically distinct regions within the images. SAM consists of three components: an image encoder, a prompt encoder, and a mask decoder. The image encoder uses a pre-trained Vision Transformer (ViT) adapted to handle high-resolution inputs [21, 22]. This encoder runs once per image and can be applied before prompting the model. It encodes the input image into a high-dimensional vector that is then used as input for the mask decoder. The prompt encoder considers two types of prompts: sparse (points, boxes, text) and dense (masks). Sparse prompts include points and boxes, which are represented by positional encodings that are summed with learned embeddings for each prompt type [23].

In contrast, text prompts are handled differently compared to other sparse prompts. Instead of using positional encodings, text prompts are embedded using the CLIP framework [24]. Dense prompts, which include masks, are embedded using convolutions and summed elementwise with the image embedding. The mask decoder maps the image embedding, prompt embeddings, and an output token to a mask. This component employs a modified Transformer decoder block and a dynamic mask prediction head [25]. The decoder block uses prompt self-attention and cross-attention in two directions (prompt-to-image embedding and vice-versa) to update all embeddings. After running two blocks, the image embedding is upsampled, and an MLP maps the output token to a dynamic linear classifier, which computes the mask foreground probability at each image location. To address ambiguity in the prompt, the model is modified to predict multiple output masks for a single prompt, with each mask having a confidence score (estimated IoU) assigned to it.

### Two-modes of IAMSAM: everything-mode and prompt-mode

As SAM can perform ROI segmentation incorporating manual prompts and automatic prompting, IAMSAM offers two main modes of operation: everything-mode and prompt-mode. In the everything-mode, the model performs image segmentation without any manual input from the user. It takes the input image and automatically generates masks for all different objects in the image. IAMSAM project segmentation masks on the tissue image with a distinct colormap, enabling users to select ROI conveniently. When the user clicks on a mask, IAMSAM captures the coordinates of the click event and adds the corresponding mask to the list of selected regions.

On the other hand, the prompt-mode allows the user to provide additional information about the model. Users can draw multiple rectangles on the main visualization panel with “*modeBarButtons.drawrect*,” the drawing tool in Plotly, by default. IAMSAM tracks the coordinates of rectangles and uses those as box prompts given to the SAM model. The model then generates the segmentation masks based on those input prompts, treating these masks as ROIs for further analysis.

### Mask filtering in the everything-mode

A mask filtering step was applied to remove unnecessary masks that do not contain any spots before visualizing the masks on the main visualization panel. Since the SAM model generates masks for the entire tissue image in the everything-mode, some masks may not have ST spots. To address this, a proportion-based filtering approach was implemented to retain only the masks that contain ST spots. The proportion of co-location between each mask and the spot coordinates was calculated by examining the overlap of mask pixels with the spot coordinates. If the calculated proportion is below 0.01, indicating a mask does not contain sufficient spots, the corresponding mask is removed from the segmentation list. This filtering process ensured that only masks containing ST spots were retained for further analysis.

### Downstream analysis

We utilized various tools and packages to extract meaningful insights from the ST data in the downstream analysis step. To identify DEGs, we employed the “*sc.tl.rank_genes_groups*” function in Scanpy, employing the Wilcoxon method. This analysis allows for the calculation of statistical significance and enables the identification of genes that exhibit significant differences in expression between conditions or cell types. Users have the flexibility to modify the cutoff values for adjusted p-value and log fold changes, enabling manual DEG definition. We specifically focus on displaying the top 10 genes with the biggest fold changes for the box plots representing DEGs. Enrichment analysis uses the “*enrichr*” function from the GSEApy package [26]. Cell type proportions of ROI 1 and ROI 2 that were calculated in the preprocessing step are shown as barplot. All visualizations in IAMSAM are created using the Plotly package.

### User interface and web application

IAMSAM is built as a Dash application, leveraging its powerful framework for creating interactive web-based data visualization and analysis tools [13]. The Dash framework, built on top of Flask, Plotly, and React, provides a highly customizable and responsive user interface. The user interface of IAMSAM is designed to provide a seamless and intuitive experience for researchers analyzing ST data. The main components of the user interface include a visualization panel with a dropdown menu to select samples to analyze, parameter panels that affect the SAM model and analysis result, and downstream analysis panels. IAMSAM offers two main modes, everything-mode, and prompt-mode, accessible through a tab menu. This allows users to easily navigate between the modes. Being a web application, IAMSAM takes advantage of various interactive features to enhance user interaction and data exploration. Users can dynamically adjust parameters, such as fold change and p-value cutoff, to customize the analysis results. The visualizations, such as volcano plots and box plots, are interactive and allow users to zoom in, zoom out, and capture specific ROI for further examination.

## Supplementary Information

Additional file 1: Supplementary Figures. Description: This file contains supplementary figures for the manuscript, including Fig S1, S2, S3 and S4.

Additional file 2: Review_history. Description: This file contains the reviewer reports and the authors’ responses.

## Data Availability

The datasets analyzed during the current study are available in the 10x Genomics repository: Human breast cancer block A Sect. 1.1 dataset [27], Human breast cancer ductal carcinoma in situ and invasive carcinoma FFPE dataset [28], Human prostate cancer adenocarcinoma with invasive carcinoma FFPE dataset [29] and Xenium Human colon preview data [30]. The mouse colon Visium data provided in the demo is available from the Gene Expression Omnibus (GEO) under the accession number GSM5213483 [31]. The code for IAMSAM is publicly available at the GitHub repository [32] and Zenodo [33] under the Apache License 2.0. Users who want to try IAMSAM without installing the code can access a demo at (https://iamsam.portrai.io).
