# Supplementary material for: IAMSAM: image-based analysis of molecular signatures using the Segment Anything Model
Source: Genome Biol. 2024 Nov 11;25:290. doi: 10.1186/s13059-024-03380-x (PMC11552325; doi:10.1186/s13059-024-03380-x)
Supplement: Supplementary file 1 — Additional file 1: Supplementary Figures. Description: This file contains supplementary figures for the manuscript, including Fig S1, S2, S3 and S4. [file 13059_2024_3380_MOESM1_ESM.docx]

IAMSAM: Image-based analysis of molecular signatures using the Segment Anything Model

Supplementary Figures

**
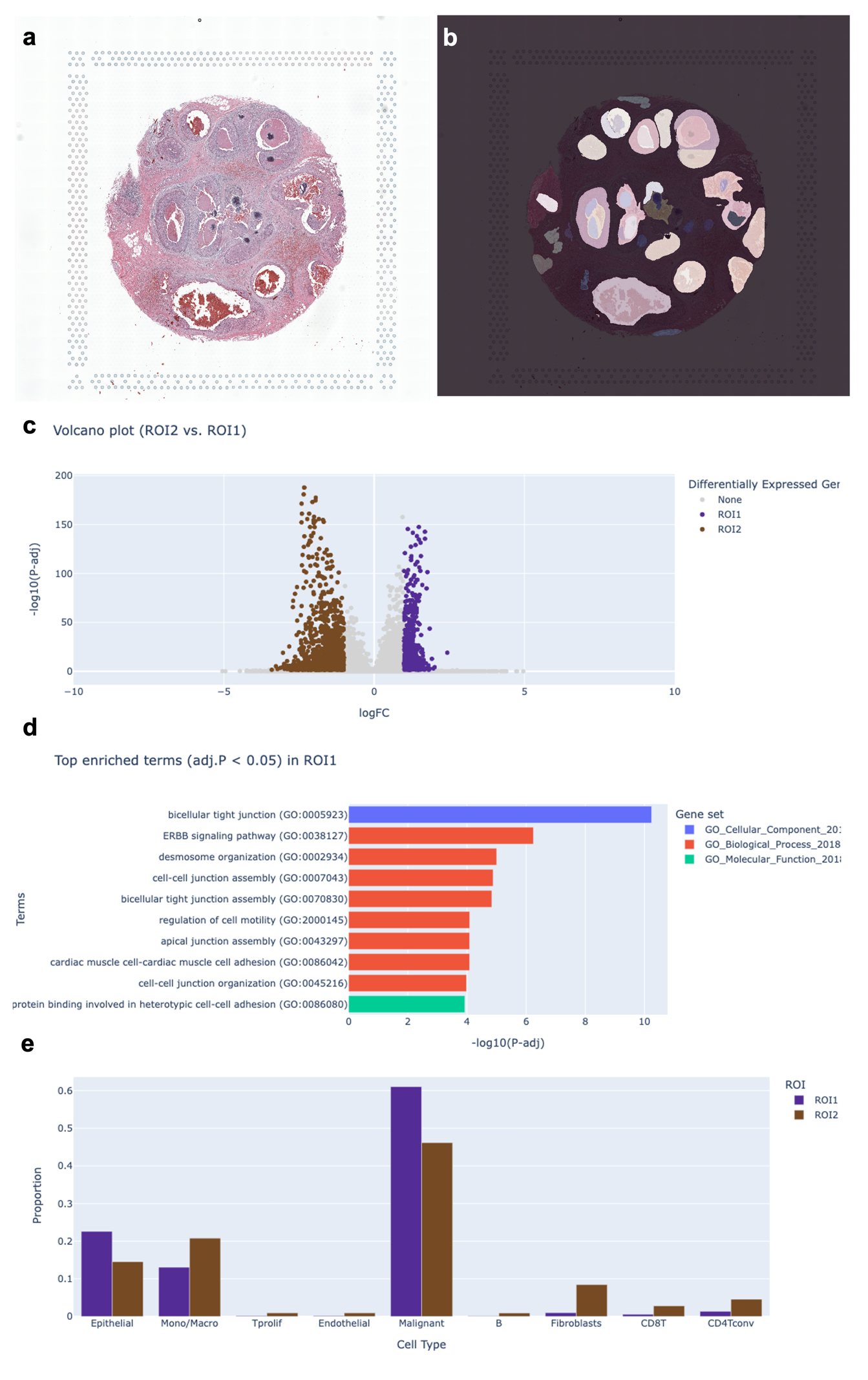
**

**Fig. S1 | Analysis of invasive carcinoma regions in the FFPE slice of human breast cancer**

(a) H&E-stained image from the ST data. (b) Masks for ROI 1 selected using everything-mode with mask confidence threshold of 0.9. Regions identified as invasive carcinoma based on formal pathology evaluation. (c) Volcano plot comparing ROI 1 (invasive carcinoma) versus ROI 2 (others) with a log fold change (log FC) threshold of 0.5 and an adjusted p-value threshold of 0.05. (d) Top enriched Gene Ontology (GO) terms for all up-regulated differentially expressed genes (DEGs) in the ROIs. (e) Cell type proportions in ROI 1 and ROI 2.


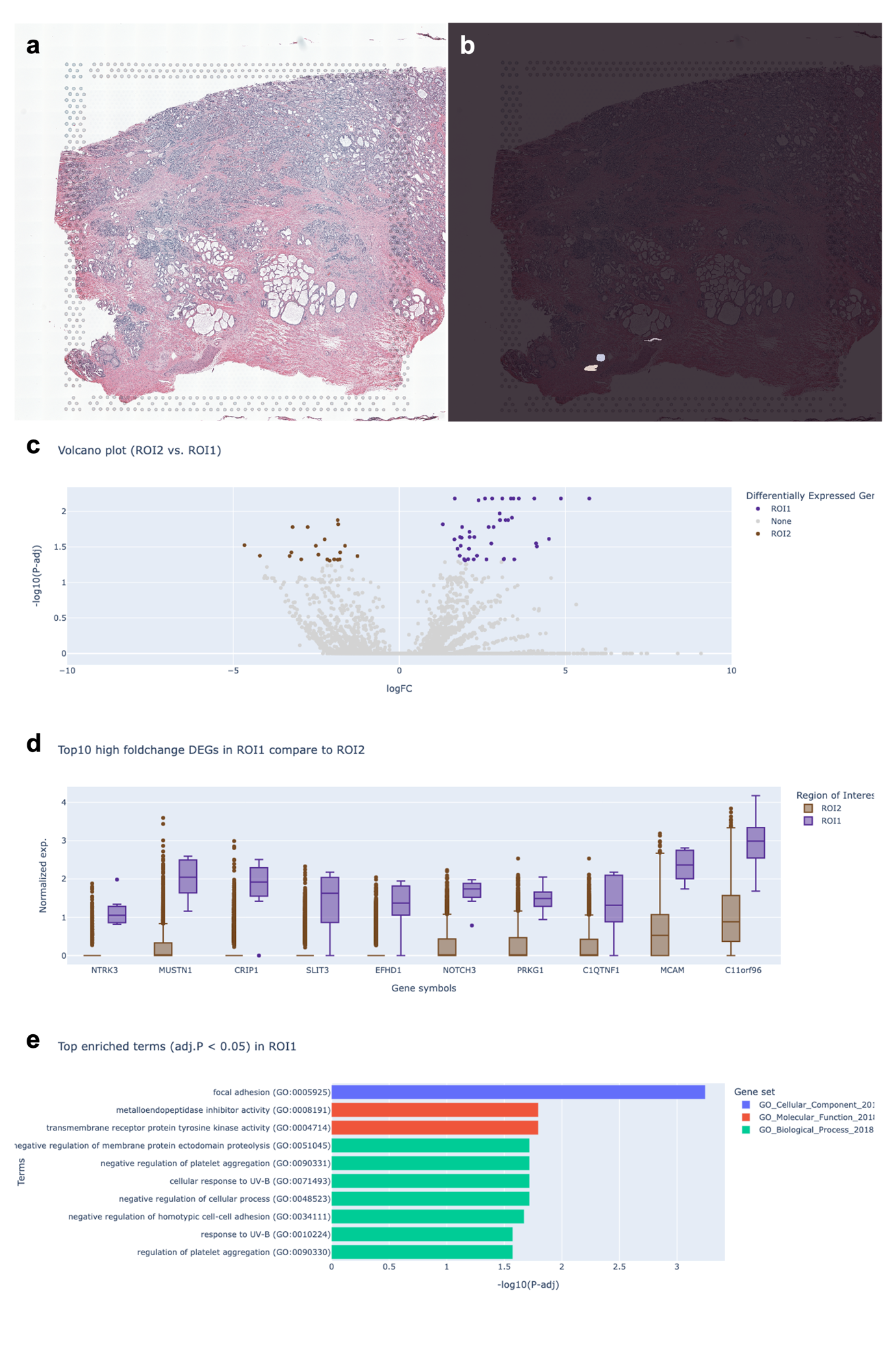


**Fig. S2 | Analysis of vessels in the FFPE slice of human prostate cancer**

(a) The H&E stained image from ST data. (b) The selection of vessels was performed using the prompt-mode in conjunction with a zoom-in interface, with validation from a pathologist to ensure accurate identification. (c) Volcano plot comparing ROI 1 (vessels) versus ROI 2 (others) with a log fold change (log FC) threshold of 0.5 and an adjusted p-value threshold of 0.05. (d) Top 10 high genes in the ROI 1 (adjusted p-value < 0.05; ordered by log FC) compared with other regions, shown as box plots. (e) Top enriched Gene Ontology (GO) terms for all up-regulated differentially expressed genes (DEGs) in the ROI 1.

**
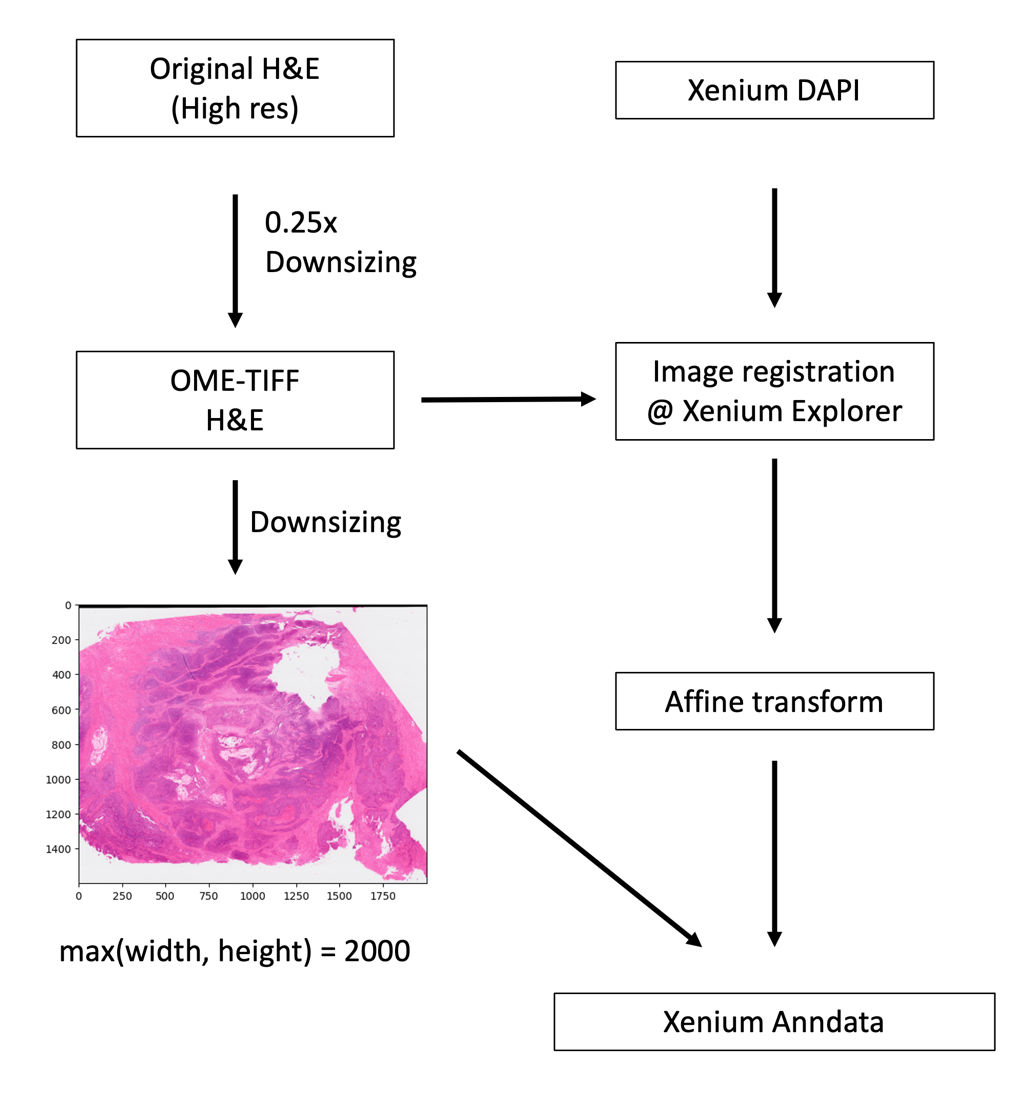
**

**Fig. S3 | Diagram for Xenium data preprocessing**

The diagram illustrates the workflow of preprocessing Xenium data for IAMSAM. The original high-resolution Hematoxylin and Eosin (H&E) stained image undergoes a 0.25x downscaling. The downscaled image is saved as an OME-TIFF file. The OME-TIFF H&E image is further downscaled to ensure the maximum dimension (width or height) is 2000 pixels for efficient calculation. The downscaled OME-TIFF H&E image and the Xenium DAPI image are registered using the Xenium Explorer. An affine transformation is applied to align the images accurately. The final aligned images are incorporated into Anndata for subsequent analysis.


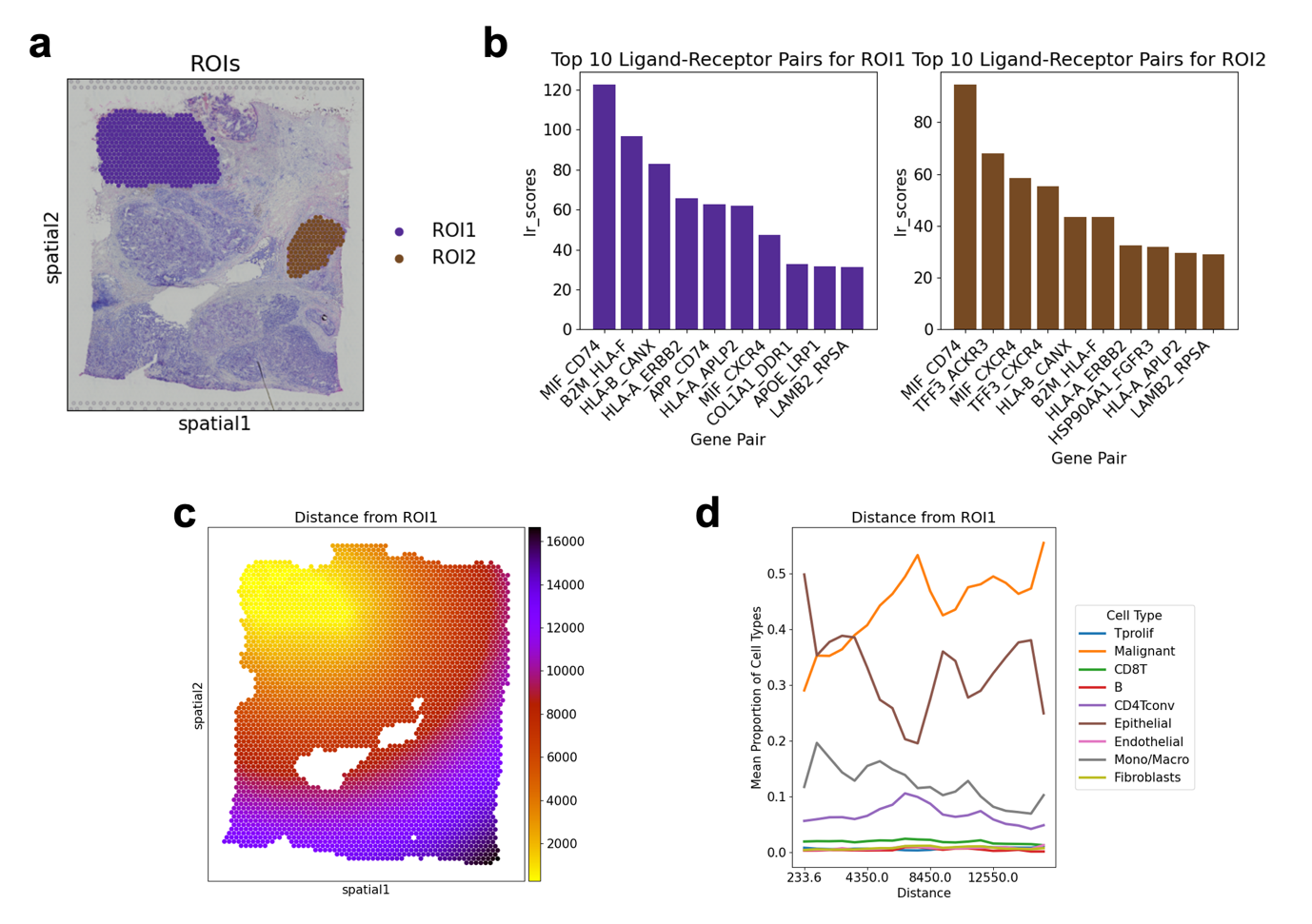


**Fig. S4 | Advanced downstream analysis beyond IAMSAM**

**(a)** ROIs selected by the IAMSAM algorithm in a human breast cancer tissue section (Block A). The figure shows two distinct ROIs, labeled as ROI1 (purple) and ROI2 (brown), overlaid on the spatial transcriptomics data. **(b)** Top 10 ligand-receptor gene pairs for each ROI, identified using the stLearn package. The left panel displays the top pairs for ROI1, while the right panel shows those for ROI2. The gene pairs are ranked by their interaction scores (lr_scores). **(c)** A spatial plot visualizing the distance from ROI1 to each spot in the tissue section. **(d)** Mean proportion of various cell types as a function of distance from ROI1.
