# Supplementary material for: IAMSAM: image-based analysis of molecular signatures using the Segment Anything Model
Source: Genome Biol. 2024 Nov 11;25:290. doi: 10.1186/s13059-024-03380-x (PMC11552325; doi:10.1186/s13059-024-03380-x)
Supplement: Supplementary file 2 — Additional file 2: Review_history. Description: This file contains the reviewer reports and the authors’ responses. [file 13059_2024_3380_MOESM2_ESM.docx]

**1st round**

**Reviewer 1**

In this article, the authors use the segment anything model to segment H&E slides of cancer, which are spatially matched with spatial transcriptomics obtained with the Visium platform. They integrate the segment anything model by Meta at the core of their approach and seem to obtain good results. It is very good to use this model because the SAM model is indeed the state-of-the-art for any image segmentation and its quality is much better than any model that any academic research groups could train by itself. Therefore, integrating this publicly available model for academic use cases could be very helpful for science. The tool presented by the authors could enable researchers to use this cutting-edge model and this could indeed be very very beneficial. So I'm very positive about the author's approach.


However, I see two problems here. First, this is not really a research article. The result section is just a description of the capabilities of their pipeline, just a description of the features. This is not scientific enough. In order to have it as a scientific article, the authors should have to present several use cases with quantifiable results comparing the findings of their pipeline to conventional methods. So the authors could either add this substantial experimental evidence in several use cases with quantitative evaluation or they could rewrite the article as a protocol article.

However, I'm not sure if this journal is open to having such purely descriptive protocol articles. Certainly, some bioinformatics journals do have sections just for methods description. My other concern is that I was not able to play around with this tool myself because I don't have any suitable data. So in any case, the authors need to provide links to data sets that can be evaluated with the model in order to enable others to play around with that easily.

**Reviewer 2**

This study introduces IAMSAM, a user-friendly web application designed for segmenting H&E/DAPI/immunofluorescence images, selecting regions of interest (ROIs), and conducting downstream analyses on spatial transcriptomics (ST) data, including differential gene expression (DEG) analysis, enrichment analysis, and cell type prediction. The application, powered by Meta's Segmentation-anything algorithm (SAM), supports various image types and simplifies manual ROI annotation within the H&E image.

IAMSAM is attempting to bridge an important gap in the current ST analysis workflow-allowing user-friendly data exploration-and it is easy to imagine that an audience of clinical pathologists would appreciate a lightweight tool to apply annotations to ST data in a systematic fashion. The application is well-crafted and user-friendly, and installation and launch processes are straightforward, with no issues running the example data. The downstream analyses are quite limited, but that could be acceptable for a tool designed primarily for data exploration.

However, the work feels incomplete. As detailed below, IAMSAM itself suffers from shortcomings that limit its utility and impact, and the paper does not make a strong case for applying SAM to ST data. There is also a serious methodological flaw in the inappropriate application of cell type prediction.

Major issues

1. CellTypist is inappropriately applied and must be removed/changed. It is a tool for annotating single-cell data but appears to be applied directly to the large Visium spots, which pool the results from many cells. Thus, CellTypist results are only likely to be accurate in ROIs of a homogenous cell type, and prone to produce artifacts in other ROIs. Because the results are displayed as a pie chart, a naïve user would easily be misled into trusting these results. It would be much better if a cell-type deconvolution approach was tied in instead.
2. I understand that the accuracy of segmentation depends on the user-defined IOU value, but how do the IAMSAM masks compare to ground truth in the first place? More generally, no attempt is made to justify the use of SAM. How well does it perform relative to other approaches? Relative to expert pathologist annotation? Take for example figure 4b--it appears that Leiden clustering based on gene expression gives a more refined segmentation than IAMSAM. Benchmarking analyses against several other approaches would be necessary before the work can be published.
3. The data export functionality doesn't provide ROIs, only the IDs and DEGs of selected regions. As a data exploration tool, IAMSAM does not attempt to perform any spatial analysis beyond ROI selection. However, it appears that these ROIs can't be exported to a different analysis package which can perform data set integration and more sophisticated spatial differential expression analyses, there's little motivation for the user to use IAMSAM.
4. Similarly, there is no mode to batch-process images. One of the primary motivations for a computer-assisted ROI selection is for reproducibility across fields of view and different data sets. This should be implemented and then exhaustively tested. For example, how stable are the results to variation in H&E staining intensity? Would a different image size or number of images affect the results?

Other issues

1. The use of the IOU metric usually assumes the availability of ground truth data for validation, which is not the case in IAMSAM. A more interpretable metric, or an explanation of how model parameters influence segmentation results, could provide clearer insights into IAMSAM's performance.
2. Image loading times can be lengthy; for instance, loading a 4.8MB high-resolution mouse brain H&E image took approximately 1 minute and 27 seconds on a MacBook Pro. This aspect of the application may benefit from optimization.
3. Enabling the export of results from enrichment analysis and cell type predictions, in addition to ROI barcode and DEG tables, would greatly enhance user convenience.
4. It would be beneficial if IAMSAM allowed for comparisons between different selected ROIs, rather than just between selected ROIs and non-ROI areas.
5. The current method for selecting ROIs is somewhat restrictive. Allowing users to adjust ROIs by resizing or moving them could significantly improve functionality.
6. Expanding IAMSAM to support images from spatial transcriptomics platforms like Xenium and MERSCOPE could make it a more versatile tool for the scientific community. However, I recognize that this would open the door to additional questions (i.e. segmenting single cells), and the feature should not be a requirement.

Minor Concerns

1. The manuscript occasionally repeats content. To improve readability, consider condensing or rephrasing repetitive sections. For example, after initially defining the "Segmentation-anything model" as SAM, consistently use the acronym thereafter instead of spelling it out 5-6 times.
2. The text should specify what is meant by "configuring multiple parameters…" for clarity.
3. "Segmentation" in the field of ST usually means identifying single cells, which is not what this manuscript tries to do. I recommend choosing different terminology (labeling? Annotation? ROI identification?)

**Reviewer 3**

This study presents a new tool called IAMSAM for analyzing spatial transcriptomics data. It enables tissue segmentation using the Segment-anything model, allowing for the semi-automatic selection of regions of interest based on morphological characteristics. This facilitates downstream analyses across selected regions, including the identification of differentially expressed genes, GO enrichment analysis, and cell type prediction. The website, as demonstrated, is user-friendly and convenient for basic transcriptomics analyses of specific regions within the image.

The methodological contribution of the study is marginal, relying on the direct adoption of the Segment Anything Model (SAM), a new AI model from Meta AI capable of "cutting out" any object in any image with a single click. SAM is a promptable segmentation system with zero-shot generalization to unfamiliar objects and images, eliminating the need for additional training. Hence, the application of SAM to tissue images is straightforward without the necessity for fine-tuning. Also, the scope of downstream analyses is limited.

How does this approach compare with existing methods, specifically for cell-level segmentation? For instance, see:

https://www.nature.com/articles/s41467-023-44560-w
https://www.nature.com/articles/s41592-023-01939-3

Would it be possible to map spatial data to single-cell RNA-seq (scRNA-seq) data?

Furthermore, is it feasible to export expression data in the region of interest for more complex analyses, such as predictions of cell-cell communication?

CellTypist is utilized to deconvolute cell types in the region of interest based on the user-selected reference model. Could you elaborate on how the outcomes could be leveraged for further analyses?

It is unclear how or where the performance of CellTypist is assessed, as mentioned in reference 14.

**Authors’ response**

Reviewer #1:

In this article, the authors use the segment anything model to segment H&E slides of cancer, which are spatially matched with spatial transcriptomics obtained with the Visium platform. They integrate the segment anything model by Meta at the core of their approach and seem to obtain good results. It is very good to use this model because the SAM model is indeed the state-of-the-art for any image segmentation and its quality is much better than any model that any academic research groups could train by itself. Therefore, integrating this publicly available model for academic use cases could be very helpful for science. The tool presented by the authors could enable researchers to use this cutting-edge model and this could indeed be very very beneficial. So I'm very positive about the author's approach.

However, I see two problems here. First, this is not really a research article. The result section is just a description of the capabilities of their pipeline, just a description of the features. This is not scientific enough. In order to have it as a scientific article, the authors should have to present several use cases with quantifiable results comparing the findings of their pipeline to conventional methods. So the authors could either add this substantial experimental evidence in several use cases with quantitative evaluation or they could rewrite the article as a protocol article.

However, I'm not sure if this journal is open to having such purely descriptive protocol articles. Certainly, some bioinformatics journals do have sections just for methods description. My other concern is that I was not able to play around with this tool myself because I don't have any suitable data. So in any case, the authors need to provide links to data sets that can be evaluated with the model in order to enable others to play around with that easily.

**Response:** We appreciate Reviewer #1's positive feedback on our approach and the potential benefits of integrating the Segment Anything Model (SAM) into our pipeline. We understand the reviewer's concerns regarding the article's format and content.

Regarding the first concern, we acknowledge that our manuscript might be more appropriate for the 'software' section rather than the traditional format of a research article. As a ‘Software’ article of the journal, we provided a detailed description of our pipeline and its capabilities. We also provided links to sample datasets and demo sites, allowing others to easily test and evaluate our tool.

Additionally, we included another section featuring an example case where IAMSAM could provide specialized analytic workflows from spatial transcriptomics data. More specifically, we conducted a deeper analysis using IAMSAM to characterize the spatial heterogeneity of tumor portions in a breast cancer sample. This approach can help uncover important molecular features associated with spatial patterns and morphology-related molecular information.

Regarding the comparison with conventional methods, we detailed the novel workflows of IAMSAM in contrast to traditional approaches that rely on morphological patterns and region-of-interest-based analyses. A dedicated subsection has been added to discuss these differences comprehensively.

To address the second concern, we are happy to provide access to our code and sample data on Github and Zenodo. Additionally, we have a hosted demo site that allows users to explore the tool without requiring their own data. We believe that by providing these resources, we can facilitate the adoption and evaluation of our pipeline by the scientific community.

Thank you for your feedback, and we hope to address your concerns in our revised manuscript.

**Reference in manuscript (p.8 Results):**

***Versatility and Expanded Capabilities of IAMSAM***

To uncover microscopic histological features, the prompt-mode in IAMSAM can be particularly powerful, especially when used with magnification. When applying IAMSAM to human prostate cancer Visium data, we demonstrated its capability to identify and select microvessels as ROIs using the prompt-mode and the zoom-in interface **(Figure 3c, Supplementary Figure 2).** Zooming in on specific tissue areas helped identify microvessels, which may not be readily apparent on a larger scale. Furthermore, our analysis revealed that pan-endothelial cell markers, such as *CAV1* (log FC = 3.21, -log10 P-adj = 2.93), *CAV2* (log FC = 2.14, -log10 P-adj = 1.56), and *CAVIN1* (log FC = 1.50, -log10 P-adj = 1.94), were up-regulated within the ROIs. In line with these findings, a GO term related to ‘focal adhesion,’ specific to endothelial cells, was enriched, indicating the involvement of endothelial cells in these ROIs (14). We also validated these microvessel areas with pathologists to ensure the accuracy of our identification.

Although IAMSAM is designed for analyzing Visium data, it can also process image-based ST technologies like Xenium and MERSCOPE if proper preprocessing steps are executed. Expanding IAMSAM to include image-based ST data allows for a broader range of applications and greater flexibility in analyzing different types of ST datasets. We demonstrated the capability of IAMSAM to analyze Xenium data using the publicly available Xenium human colon cancer dataset. If a post-Xenium H&E image is available, users can preprocess Xenium data with affine transformation and resizing **(Figure 3d, Supplementary Figure 3).** This expansion enhances the versatility of IAMSAM, making it a powerful tool for integrating and analyzing ST data from various sources. Lastly, we explored the application of IAMSAM with an optical image different from H&E staining **(Figure 3e)**. We utilized a combined image of three distinct color channels corresponding to DAPI (4’,6-diamidino-2-phenylindole), Anti-GFAP, and Anti-NeuN staining. In this case, the successful identification of the Dentate Gyrus (DG) structure demonstrated the versatility and feasibility of IAMSAM in handling different imaging modalities. This finding further solidifies SAM as a general image segmentation algorithm that can be applied across various experimental setups. This feature highlights the broad applicability of IAMSAM and its potential to provide valuable information from diverse imaging modalities that spatially correspond to ST data (15).

***Characterizing Spatial Tumor Heterogeneity in a Breast Cancer Sample Using IAMSAM***

To demonstrate an example of IAMSAM to discover the finding by integrating ST with morphological features, we inspected cancer heterogeneity within a Human breast cancer block A section 1.1 dataset. We selected two ROIs **(Figure 4a-d)** based on distinct morphological features observed in the dataset as an automatic method for delineating morphologically characteristic regions based on IAMSAM. Notably, ROI1 is identified as an invasive region, while ROI2 is classified as a ductal carcinoma in situ (DCIS) portion according to the pathological annotation of the previous literature (16). The IAMSAM analysis identified ROI 1 as primarily characterized by immune-related processes compared to ROI 2. Differential gene expression analysis identified top genes such as *PLA2G2A*, *GPR143*, *LINC00052*, *UNC5C*, and *PLA2G2D* as significantly upregulated in ROI 1 compared to ROI 2 **(Figure 4e)**. Enrichment analysis revealed terms such as MHC protein complex, cellular response to interferon-gamma, and cytokine-mediated signaling pathway **(Figure 4f)**. These enrichments suggest a significant presence of immune cell infiltration and activity within ROI 1. The cell type proportion analysis further supported this, showing a high presence of immune cells such as monocytes/macrophages and CD4 T-cells **(Figure 4i)**. These findings align with the characteristics of Invasive Ductal Carcinoma (IDC), where immune interactions are progressed compared with DCIS (17,18). In contrast to ROI 1, genes such as *CEACAM1*, *TGFBR1*, *ZNF737*, and *PLK2* were significantly upregulated in ROI 2 **(Figure 4g)**. The enriched GO terms for ROI 2 included epidermis development, cell-substrate junction assembly, and hemidesmosome assembly, which are indicative of epithelial processes and cell adhesion **(Figure 4h)**. The cell type proportion analysis revealed a predominance of epithelial cells and malignant cells, consistent with the features of the tumor core where malignant cells are predominant and exhibit strong epithelial characteristics **(Figure 4i)**. This result aligns with previous histological annotations (16), which identified ROI2 as the tumor region of DCIS and ROI1 as the invasive region of breast cancer. This analysis highlights IAMSAM's capability to effectively analyze heterogeneous tumor samples without requiring manual histological annotation and subjective delineation, thus offering significant potential for cancer researchers.

***Workflow Advantages of IAMSAM Over Traditional Methods in Analyzing Spatial Heterogeneity***

The workflow of IAMSAM in analyzing the spatial morphological heterogeneity surpasses that of traditional methods, as illustrated in Figure 5. Traditional methods involve a manual and time-consuming workflow, requiring multiple steps and tools. Typically, for Visium data, a loupe file is examined using Loupe Browser, where ROIs are manually drawn from scratch **(Figure 5a)**. Subsequently, users must export the barcode table and match it with the original matrix data, subjecting it to downstream bioinformatic analysis using R or Python. The entire process typically requires a considerable amount of time and involves manual steps, particularly in delineating ROIs. This introduces limitations in reproducibility, which can vary depending on the complexity of the sample and the proficiency of the analyst. In contrast, IAMSAM offers a streamlined and automated workflow that leverages morphological features for analysis **(Figure 5b)**. The preprocessing script transforms the data into an AnnData file, which is then analyzed using IAMSAM. With box prompting, IAMSAM not only simplifies the process but also makes it easily repeatable multiple times, allowing for the inspection of various regions with consistent and accurate identification of ROIs. The downstream analysis is integrated within the IAMSAM framework, significantly reducing the entire time. IAMSAM's automated approach minimizes manual effort and reduces the potential for human error, ensuring more reliable and reproducible results. This streamlined workflow highlights the efficiency and accuracy of IAMSAM, making it a superior alternative to traditional methods for inspecting morphological heterogeneity and spatial patterns of the tissue, especially in tumor heterogeneity. The reduction in analysis time and manual intervention not only enhances productivity but also improves the consistency and reliability of the results, making IAMSAM an invaluable tool for cancer research.

**Reference in manuscript (p.16 Availability of data and materials):**

The code for IAMSAM is publicly available at the GitHub repository (<https://github.com/portrai-io/IAMSAM>) and Zenodo (<https://doi.org/10.5281/zenodo.12175539>). Users who want to try IAMSAM without installing the code can access a demo at ([https://iamsam.portrai.io](https://iamsam.portrai.io/)).

Reviewer #2

This study introduces IAMSAM, a user-friendly web application designed for segmenting H&E/DAPI/immunofluorescence images, selecting regions of interest (ROIs), and conducting downstream analyses on spatial transcriptomics (ST) data, including differential gene expression (DEG) analysis, enrichment analysis, and cell type prediction. The application, powered by Meta's Segmentation-anything algorithm (SAM), supports various image types and simplifies manual ROI annotation within the H&E image.

IAMSAM is attempting to bridge an important gap in the current ST analysis workflow-allowing user-friendly data exploration-and it is easy to imagine that an audience of clinical pathologists would appreciate a lightweight tool to apply annotations to ST data in a systematic fashion. The application is well-crafted and user-friendly, and installation and launch processes are straightforward, with no issues running the example data. The downstream analyses are quite limited, but that could be acceptable for a tool designed primarily for data exploration.

However, the work feels incomplete. As detailed below, IAMSAM itself suffers from shortcomings that limit its utility and impact, and the paper does not make a strong case for applying SAM to ST data. There is also a serious methodological flaw in the inappropriate application of cell type prediction.

**Major issues**

1. CellTypist is inappropriately applied and must be removed/changed. It is a tool for annotating single-cell data but appears to be applied directly to the large Visium spots, which pool the results from many cells. Thus, CellTypist results are only likely to be accurate in ROIs of a homogenous cell type, and prone to produce artifacts in other ROIs. Because the results are displayed as a pie chart, a naïve user would easily be misled into trusting these results. It would be much better if a cell-type deconvolution approach was tied in instead.

**Response:** We appreciate the reviewer's comment and agree that applying CellTypist directly to Visium data may not be the most appropriate approach. We understand that our intention was to provide a simple and user-friendly method, but we agree that using cell type deconvolution methods is a more reliable approach for analyzing Visium data.

In response to the reviewer's comment, we have modified our manuscript and code to visualize the results from cell type deconvolution analysis after preprocessing steps. To facilitate this step, we have also provided notebooks for users to preprocess and perform cell type deconvolution analysis on their own (They are updated in the github as a preprocess step). This allows users to have more control over the analysis and to customize it to their specific needs. For example, cell typing or deconvolution methods could be changed according to the user’s preference. This approach allows for a more accurate and reliable estimation of cell type proportions in each ROI. We believe that this modification will provide a more robust and accurate analysis of the data. We would like to thank the reviewer for bringing this to our attention and for helping us to improve the quality and accuracy of our analysis.

**Reference in manuscript (p.6 Results)**: For the last panel, IAMSAM provides cell type proportion within the selected ROIs. We exploit CellDART [13] to annotate Visium data with reference scRNAseq data by default, but users can also choose other cell type deconvolution algorithms in the preprocessing step. The proportions of cell types are visualized as a bar chart, displaying the differences between ROI 1 and ROI 2 for clarity and simplicity. This concise representation offers a clear overview of the predominant cell types present in the tissue sample and aids in understanding the cellular composition within the spatial context.

2. I understand that the accuracy of segmentation depends on the user-defined IOU value, but how do the IAMSAM masks compare to ground truth in the first place? More generally, no attempt is made to justify the use of SAM. How well does it perform relative to other approaches? Relative to expert pathologist annotation? Take for example figure 4b--it appears that Leiden clustering based on gene expression gives a more refined segmentation than IAMSAM. Benchmarking analyses against several other approaches would be necessary before the work can be published.

**Response :** We appreciate the reviewer's comment regarding the comparison of IAMSAM masks to ground truth. As the reviewer commented, it was true that segmentation depends on their IOU value, but it is actually predicted-IOU-threshold that evaluate the confidence of predicted mask. So we did not make any ground truth mask for this tasks. Therefore, the 'IOU value' serves as a hyperparameter that determines the degree of fineness or coarseness of the SAM algorithm.

Regarding the justification for using SAM, we acknowledge that our original manuscript did not provide sufficient context. SAM has been widely used in the field of image segmentation due to its ability to capture spatial relationships between pixels [1]. In our study, we chose SAM because of its ability to incorporate spatial information and adapt to varying tissue morphology. Clustering methods such as Leiden clustering are effective for analysis; however, it relies solely on gene expression data and does not incorporate the combined image data of spatial transcriptomics (ST). Therefore, the primary purpose and justification for using SAM is to integrate image data and its segmented results with gene expression data.We have added a paragraph to the manuscript to provide a brief overview of the advantages of SAM and its relevance to our study.

Regarding Leiden clustering, we acknowledge that Leiden clustering based on gene expression appears to provide a more refined segmentation than IAMSAM. However, we would like to clarify that IAMSAM is designed to segment spatially coherent regions based on morphological features, whereas Leiden clustering is a clustering algorithm that groups cells based on gene expression profiles. As mentioned above, the purpose of SAM is to use image-based segmentation to analyze ST data. The two methods are not directly comparable, and each has its own strengths and limitations.

**Reference in manuscript (Page 3, Introduction):** We used this model to handle various tissue images (e.g., H&E, DAPI, and immunofluorescence images), taking advantage of its effectiveness and adaptability to handle different image distributions and workloads through zero-shot or few-shot learning. This excellency leads to conducting various downstream analyses such as identifying differentially expressed genes (DEGs), enrichment analysis, and cell type prediction of user-selected regions. Moreover, the regions can be determined by image patterns rather than gene expression, providing an opportunity to analyze gene expression patterns and features based on image-based key patterns.

**Reference in manuscript** **(Page 10, Discussion):** Integrating SAM into ST libraries has shown great promise as a new workflow to enhance the interpretation of image-based characteristics in ST data analysis, leading to a more comprehensive molecular interpretation. This integration allows for accurate segmentation of histologically distinct structures and produces segments that align closely with gene expression clusters.

3. The data export functionality doesn't provide ROIs, only the IDs and DEGs of selected regions. As a data exploration tool, IAMSAM does not attempt to perform any spatial analysis beyond ROI selection. However, it appears that these ROIs can't be exported to a different analysis package which can perform data set integration and more sophisticated spatial differential expression analyses, there's little motivation for the user to use IAMSAM.

**Response:** We understand the concern that the current data export functionality of IAMSAM only provides IDs and DEGs, without providing the actual ROIs. In response to the reviewers' comment, we add functionality to export ROI annotated *Anndata* file, allowing users to export the selected regions for further analysis in other packages. This will enable users to leverage the strengths of IAMSAM for ROI selection, while integrating the results with other tools for more advanced spatial analysis.

4. Similarly, there is no mode to batch-process images. One of the primary motivations for a computer-assisted ROI selection is for reproducibility across fields of view and different data sets. This should be implemented and then exhaustively tested. For example, how stable are the results to variation in H&E staining intensity? Would a different image size or number of images affect the results?

**Response:** We thank the reviewer for pointing out the important point. We acknowledge the limitations of SAM, particularly regarding its ability to achieve consistent segmentation results across multiple images. While batch processing may not currently be feasible with SAM, we believe the tool remains valuable for identifying meaningful regions of interest within a single dataset. Since many ST data are obtained from different samples or disease conditions, analyzing each sample individually is crucial. Most analytic tools are therefore designed to facilitate in-depth analysis of each single sample. To address this concern, we will include a brief discussion in the manuscript highlighting this limitation and suggesting areas for future improvement, such as enhancing the tool's robustness to variations in H&E staining intensity and exploring the impact of different image sizes and quantities on the results.

**Reference in manuscript (Page 10, Discussion):** Despite the numerous advantages of IAMSAM in identifying meaningful regions of interest within ST data, some limitations warrant further discussion and future improvement. Firstly, IAMSAM currently does not support processing of multiple images. One of the primary motivations for computer-assisted ROI selection is to ensure reproducibility across different fields of view and datasets. However, the segmentation process with SAM could be varied in multiple images due to batch effects of images. This limitation underscores the need for developing and integrating unified processing capabilities of segmentation to enhance efficiency, especially in large-scale studies. In this regard, the stability of SAM's segmentation results may be influenced by variations in H&E staining intensity. Differences in staining intensity can impact the accuracy of segmentation, potentially leading to inconsistent results. Future iterations of IAMSAM should focus on improving the robustness of tool to such variations. This could involve the implementation of normalization techniques or adaptive algorithms that adjust to staining intensity changes, ensuring more reliable segmentation outcomes. Exploring the impact of varying image dimensions and the number of images on segmentation performance can be a future direction for the applicability of this tool. Future developments should aim to assess and optimize the performance across a range of image sizes and dataset volumes.

**Other issues**

1. The use of the IOU metric usually assumes the availability of ground truth data for validation, which is not the case in IAMSAM. A more interpretable metric, or an explanation of how model parameters influence segmentation results, could provide clearer insights into IAMSAM's performance.

**Response:** The IOU score mentioned refers to the confidence level of the masks predicted by the SAM model, indicating the IoU prediction, which differs from ground truth validation [2]. In IAMSAM, adjusting the IOU score threshold controls the level of detail in the predicted masks, allowing for either coarse or fine adjustments.

**Reference in manuscript (Page 6, Results):** The 'Mask confidence threshold' parameter (Figure 3a) is a crucial factor for users to consider because it determines the threshold value used to decide whether a predicted object or region in an image is considered a true positive or not. It is described as intersection-over-union (IOU) score in original literature, which is a metric used to measure the overlap between the predicted segmentation mask and the ground truth mask during training.

2. Image loading times can be lengthy; for instance, loading a 4.8MB high-resolution mouse brain H&E image took approximately 1 minute and 27 seconds on a MacBook Pro. This aspect of the application may benefit from optimization.

**Response:** Thank you for your feedback regarding the image loading times. The demo website operates on instances with limited performance, which may inherently restrict its efficiency. To address this, we recommend running the code from our GitHub repository on a server with GPU capabilities. Compared with the Demo site, this setup should provide better performance and more efficient handling of the user's own data. We will include this suggestion in our manuscript to guide users towards achieving optimal results.

3. Enabling the export of results from enrichment analysis and cell type predictions, in addition to ROI barcode and DEG tables, would greatly enhance user convenience.

**Response:** We understand the importance of enabling the export of results from enrichment analysis and cell type predictions, in addition to ROI barcode and DEG tables, to enhance user convenience. In response to the reviewers' comment, we have added functionality to export ROI-annotated CSV files, allowing users to efficiently merge the information into metadata for further analysis in other packages such as Seurat or Scanpy. Additionally, we have introduced 'Download plot as a PNG' buttons, enabling users to easily save enrichment plots and cell type pie charts in PNG format for further analysis or presentation.

4. It would be beneficial if IAMSAM allowed for comparisons between different selected ROIs, rather than just between selected ROIs and non-ROI areas.

**Response:** We appreciate the suggestion to allow for comparisons between different selected ROIs in IAMSAM. We agree that this feature would be beneficial for users to compare and contrast different regions of interest. As a result, we have added a dropdown menu for both ROI1 and ROI2 to our application, supporting both 'selected ROI vs the others' and 'ROI1 vs ROI2' comparisons. When ROI2 is empty, it is automatically handled for ROI1 vs the others comparison. We have also adjusted the DEG, enrichment analysis, and cell type deconvolution analyses to accommodate this new feature.

**Reference in manuscript (Page 4. Results):** In the everything-mode, IAMSAM automatically generates segment masks based on morphological features along whole tissues. On the other hand, the prompt-mode allows users to draw rectangle boxes, which serve as input prompts for the SAM model. Afterwards, users have the option to select one or multiple masks for ROI 1 and ROI 2 from the mask lists before proceeding with downstream analysis. IAMSAM automatically extracts the gene expression profile from the chosen ROIs, identifying not only DEGs between the ROIs

**Reference in manuscript (Page 5. Results):** Genes that meet the criteria of having a fold change value exceeding the FC cutoff and an adjusted p-value less than the adjusted p-value cutoff are displayed in purple for ROI 1 and brown for ROI 2, while the remaining genes are shown in gray. The box plot, on the other hand, focuses on the top 10 genes selected from the up-regulated DEGs within the ROI 1. These genes are ranked based on their fold changes, reflecting the relative difference in expression levels between the ROI 1 and ROI 2.

5. The current method for selecting ROIs is somewhat restrictive. Allowing users to adjust ROIs by resizing or moving them could significantly improve functionality.

**Response:** Thanks for your suggestion to improve the ROI selection process by allowing users to adjust ROIs through resizing or moving. While this could indeed enhance the functionality of IAMSAM, we would like to note that our tool is designed to streamline the process and minimize manual intervention by leveraging the model's segmentation masks based on morphological features.

However, we agree that allowing users to resize the mask could provide more flexibility for those who want to analyze the marginal areas of the ROI. To address this, we have added the mask modulator functionality to IAMSAM, which enables dilation and erosion of the predicted mask. This feature allows users to adjust the mask boundaries to suit their specific needs.

6. Expanding IAMSAM to support images from spatial transcriptomics platforms like Xenium and MERSCOPE could make it a more versatile tool for the scientific community. However, I recognize that this would open the door to additional questions (i.e. segmenting single cells), and the feature should not be a requirement.

**Response:** We appreciate the suggestion to expand IAMSAM to support images from spatial transcriptomics platforms like Xenium and MERSCOPE, as we recognize the potential benefits this could bring to the scientific community. As a practical solution, we are pleased to offer a notebook that preprocesses Xenium raw data, enabling its analysis in IAMSAM. This preprocessing step includes resizing H&E images, registration, and cell type annotation using the TACCO package.

**Reference in manuscript (Page 7, Results):** Although IAMSAM is designed for analyzing Visium data, it can also process image-based ST technologies like Xenium and MERSCOPE if proper preprocessing steps are executed. Expanding IAMSAM to include image-based ST data allows for a broader range of applications and greater flexibility in analyzing different types of spatial transcriptomics datasets. We demonstrated the capability of IAMSAM to analyze Xenium data using the publicly available Xenium human colon cancer dataset. If a post-Xenium H&E image is available, users can preprocess Xenium data with affine transformation and resizing (Figure 4c, Supplementary Figure 3). This expansion enhances the versatility of IAMSAM, making it a powerful tool for integrating and analyzing spatial transcriptomics data from various sources. Lastly, we explored the application of IAMSAM with an optical image different from H&E staining (Figure 4d). We utilized a combined image of three distinct color channels corresponding to DAPI (4’,6-diamidino-2-phenylindole), Anti-GFAP, and Anti-NeuN staining. In this case, the successful identification of the Dentate Gyrus (DG) structure demonstrated the versatility and feasibility of IAMSAM in handling different imaging modalities. This finding further solidifies SAM as a general image segmentation algorithm that can be applied across various experimental setups. This feature highlights the broad applicability of IAMSAM and its potential to provide valuable information from diverse imaging modalities that spatially correspond to ST data.

**Minor Concerns**

1. The manuscript occasionally repeats content. To improve readability, consider condensing or rephrasing repetitive sections. For example, after initially defining the "Segmentation-anything model" as SAM, consistently use the acronym thereafter instead of spelling it out 5-6 times.

**Response :** Thank you for pointing out the repetition in the manuscript. We will make sure to consistently use the acronym "SAM" after its initial definition, rather than spelling it out multiple times.

2. The text should specify what is meant by "configuring multiple parameters…" for clarity.

**Response :** Thank you for the feedback. We will revise the text to specify what is meant by this phrase, providing more clarity for the reader.

**Reference in manuscript (Page 4, Results):** After configuring multiple parameters, such as mask confidence threshold, mask opacity, and mask size, users can click the ‘Run SAM’ button to make inferences from the Segment-anything model (SAM).

3. "Segmentation" in the field of ST usually means identifying single cells, which is not what this manuscript tries to do. I recommend choosing different terminology (labeling? Annotation? ROI identification?)

**Response :** We agree with your concern about the word “segmentation”, which can be confused in the field of spatial transcriptomics, referring to cell segmentation. Nonetheless, since segmentation is also widely used in computer vision and other image processing fields, we will specify this terminology as "ROI segmentation" to avoid confusion and accurately convey the manuscript's focus.

Reviewer #3

This study presents a new tool called IAMSAM for analyzing spatial transcriptomics data. It enables tissue segmentation using the Segment-anything model, allowing for the semi-automatic selection of regions of interest based on morphological characteristics. This facilitates downstream analyses across selected regions, including the identification of differentially expressed genes, GO enrichment analysis, and cell type prediction. The website, as demonstrated, is user-friendly and convenient for basic transcriptomics analyses of specific regions within the image.

The methodological contribution of the study is marginal, relying on the direct adoption of the Segment Anything Model (SAM), a new AI model from Meta AI capable of "cutting out" any object in any image with a single click. SAM is a promptable segmentation system with zero-shot generalization to unfamiliar objects and images, eliminating the need for additional training. Hence, the application of SAM to tissue images is straightforward without the necessity for fine-tuning. Also, the scope of downstream analyses is limited.

How does this approach compare with existing methods, specifically for cell-level segmentation? For instance, see:

<https://www.nature.com/articles/s41467-023-44560-w> (BIDcell)

<https://www.nature.com/articles/s41592-023-01939-3> (SCS)

Would it be possible to map spatial data to single-cell RNA-seq (scRNA-seq) data? Furthermore, is it feasible to export expression data in the region of interest for more complex analyses, such as predictions of cell-cell communication?

**Response :** We appreciate your inquiry about the comparison of our approach with existing methods, specifically for cell-level segmentation. The methods you mentioned, such as BIDcell and SCS, are indeed focused on achieving more precise cell segmentation at subcellular resolution in image-based spatial transcriptomics. In contrast, IAMSAM leverages morphological features extracted from images produced alongside spatial transcriptomics data for downstream analysis, serving a different purpose.

Regarding the integration of spatial data with single-cell RNA-seq (scRNA-seq) data, tools like Harmony and scVI enable the embedding of these data types into a unified space. This allows for the exploration of relationships between spatial and transcriptomic data.

Furthermore, IAMSAM allows for the export of cell barcodes within regions of interest (ROIs). By leveraging this capability, one can extract gene expression from two ROIs and utilize tools like CellChat to estimate cell-cell communication between these regions based on gene expression. This enables more complex analyses, such as predictions of cell-cell communication, and expands the possibilities for downstream exploration.

CellTypist is utilized to deconvolute cell types in the region of interest based on the user-selected reference model. Could you elaborate on how the outcomes could be leveraged for further analyses? It is unclear how or where the performance of CellTypist is assessed, as mentioned in reference 14.

**Response :** We appreciate your suggestion to elaborate on the outcomes of CellTypist. In response to the reviewer's comment, we have made modifications to both our manuscript and code. Specifically, we now visualize the results from cell type deconvolution analysis after preprocessing steps. While various methods for cell type deconvolution exist, in our example, we utilized the CellDART method, as developed in previous research. With cell type information within user-defined ROIs powered by IAMSAM, users can leverage this information to explore their own datasets with an AI-powered approach, enabling further analyses and discoveries.

**Reference**

[1] Deng, Ruining, et al. Segment anything model (sam) for digital pathology: Assess zero-shot segmentation on whole slide imaging. arXiv preprint arXiv. 2023.

[2] Kirillov A, Mintun E, Ravi N, Mao H, Rolland C, Gustafson L, et al. Segment Anything. arXiv preprint arXiv. 2023.

**2^nd^ round**

**Reviewer 1**

thank you. no further questions

**Reviewer 2**

The authors have addressed my concerns--congratulations on the study! Looking forward to using the tool

**Reviewer 3**

While the authors improved the manuscript during the revision, I am not convinced by the responses provided to my comments, nor do I see compelling revisions to the manuscript addressing my concerns.

Specifically, the following concerns have not been adequately addressed:

The authors need to compare their tool with existing ones and elaborate on its contributions. The reasoning provided in the response (i.e., IAMSAM leverages morphological features) is not compelling. The authors need to justify how IAMSAM addresses gaps not covered by previous methods.

The authors need to specify the usability of the tool for further downstream and more advanced analyses. For instance, they should discuss the tool's capability to extract gene expressions from ROIs and its compatibility with other tools for cell-cell communication, among other analyses. While I don't necessarily request that these analyses be performed, these capabilities should be clearly elaborated in the manuscript.

**Authors’ response**

Reviewer #3

1. The authors need to compare their tool with existing ones and elaborate on its contributions. The reasoning provided in the response (i.e., IAMSAM leverages morphological features) is not compelling. The authors need to justify how IAMSAM addresses gaps not covered by previous methods.

**Response:** Thank you for your constructive feedback, which has helped us improve our manuscript significantly. We have added a detailed comparison of IAMSAM with traditional methods, specifically highlighting how IAMSAM addresses gaps not covered by previous methods. The new paragraph emphasizes the advantages of IAMSAM over traditional methods, such as those using Loupe Browser for Visium data.

**Reference in manuscript(Results, p. 9):** This manual process is time-consuming and highly dependent on the analyst’s skill and consistency, leading to variability and reproducibility issues due to human error and subjective judgment. IAMSAM addresses this gap by automating the identification of ROIs using advanced image processing techniques that leverages morphological features (Figure 5b). The use of box prompting in IAMSAM simplifies the process and ensures consistency, allowing multiple inspections of various regions. This automation eliminates the need for manual intervention, significantly reducing the time required for ROI identification and improving reproducibility. Additionally, traditional workflows often involve multiple disjointed steps and tools, such as exporting barcode tables, matching with matrix data, and performing separate downstream analyses using R or Python. This fragmentation is inefficient and prone to errors, as each step requires separated code, increasing the overall processing time and introducing potential points of failure. IAMSAM addresses this gap by providing a seamless, end-to-end workflow that integrates data preprocessing, ROI identification, and downstream analysis within a single platform.

1. advanced analyses. For instance, they should discuss the tool's capability to extract gene expressions from ROIs and its compatibility with other tools for cell-cell communication, among other analyses. While I don't necessarily request that these analyses be performed, these capabilities should be clearly elaborated in the manuscript.

**Response:** We have expanded the manuscript to clearly outline the usability of IAMSAM for downstream analyses. This includes detailed descriptions of how IAMSAM can be used to extract gene expressions from ROIs and its compatibility with tools for cell-cell communication. We have provided examples and potential workflows to illustrate these capabilities, enhancing the clarity of our method's application.

**Reference in manuscript(Results, p. 9):** Beyond identifying distinct molecular and cellular characteristics within ROIs, IAMSAM can extend its utility by integrating with advanced bioinformatics tools for further analyses. For example, users can employ tools such as stLearn(5) to analyze cell-cell communication within ROIs selected by IAMSAM. This integration allows for the identification of top-scored ligand-receptor pairs for each ROI, providing insights into the molecular interactions within specific tissue regions (Supplementary Figure 4b). Additionally, ROIs can be inspected using compositional frameworks like TACCO(19), calculating distances from the ROI and illustrating changes in cell type deconvolution along these distances (Supplementary Figures 4c-d). This compatibility facilitates seamless integration with other tools, enabling more comprehensive and advanced analyses for researchers.

**3^rd^ round**

**Reviewer 3**

The authors have satisfactorily addressed my comments. They have provided a clear explanation of IAMSAM's usability for downstream analyses. The articulation of the added-value of IAMSAM (as a software) compared with previous tools was convincing.
